# The role of complex cues in social and reproductive plasticity

**DOI:** 10.1007/s00265-018-2539-x

**Published:** 2018-07-07

**Authors:** Alice A. Dore, Laurin McDowall, James Rouse, Amanda Bretman, Matthew J. G. Gage, Tracey Chapman

**Affiliations:** 1 School of Biological Sciences, University of East Anglia, Norwich Research Park, Norwich, NR4 7TJ UK; 2 School of Biology, Faculty of Biological Sciences, University of Leeds, Leeds, LS2 9JT UK

**Keywords:** Phenotypic plasticity, Sexual selection, Fitness, Reproductive success, Multimodal, Communication

## Abstract

Phenotypic plasticity can be a key determinant of fitness. The degree to which the expression of plasticity is adaptive relies upon the accuracy with which information about the state of the environment is integrated. This step might be particularly beneficial when environments, e.g. the social and sexual context, change rapidly. Fluctuating temporal dynamics could increase the difficulty of determining the appropriate level of expression of a plastic response. In this review, we suggest that new insights into plastic responses to the social and sexual environment (social and reproductive plasticity) may be gained by examining the role of complex cues (those comprising multiple, distinct sensory components). Such cues can enable individuals to more accurately monitor their environment in order to respond adaptively to it across the whole life course. We briefly review the hypotheses for the evolution of complex cues and then adapt these ideas to the context of social and sexual plasticity. We propose that the ability to perceive complex cues can facilitate plasticity, increase the associated fitness benefits and decrease the risk of costly ‘mismatches’ between phenotype and environment by (i) increasing the robustness of information gained from highly variable environments, (ii) fine-tuning responses by using multiple strands of information and (iii) reducing time lags in adaptive responses. We conclude by outlining areas for future research that will help to determine the interplay between complex cues and plasticity.

## Introduction

Phenotypic plasticity is the extent to which an organism with a given genotype can express alternative phenotypes under different environmental conditions (Gause 1947; Bradshaw 1965). It may represent a key component of adaptation to rapid environmental change (Agrawal 2001; Charmantier et al. 2008). In this review, we discuss how plasticity expressed in response to the social and sexual environment may be facilitated by the perception of environmental cues composed of multiple distinct components (‘complex cues’; Hebets and Papaj 2005). We focus on the socio-sexual environment, as it is likely to be both complex (involving multiple individuals) and rapidly changeable (Charmantier et al. 2008; Kasumovic et al. 2008). We identify the factors that influence the proximate expression of plastic responses to the social and sexual environment (social and reproductive plasticity) and discuss how these can ultimately affect the evolution of plasticity. Simple and complex cues are first defined within the context of social and sexual plasticity, and their potential roles across the whole life course are then discussed. We summarise current hypotheses for the evolution of complex cues and adapt these concepts to social and sexual plasticity. We propose that perceiving complex cues may facilitate plasticity and avoid costly phenotype-environment mismatches by (i) maximising information transfer in variable environments, (ii) facilitating the fine-tuning of phenotypes to the environment and (iii) reducing time lags between perception of cues and expression of plasticity.

## Simple and complex cues in assessment of the social environment

In this review, we consider a ‘cue’ as any kind of indicator that can be used to perceive information about the social or sexual environment by an individual. Such indicators can be either ‘intentionally’ or unintentionally transmitted by a signalling individual (Glossary). For instance, body size, which can potentially signal information on aspects of morphology/general condition, versus visual/auditory displays, which give potentially more targeted information, can both be considered as cues. A ‘complex cue’ comprises two or more distinct subcomponents exchanged during the course of one encounter, which is capable of inducing or influencing a response in a receiver (Hebets and Papaj 2005; Glossary). This contrasts with ‘simple cues’, in which information received is a single component. In addition, complex cue components can be perceived from one (‘unimodal’) or multiple (‘multimodal’) sensory modalities (Hebets and Papaj 2005). Complex cues would be unimodal if female choice was influenced by two or more male sexual ornaments, all processed visually (Møller and Pomiankowski 1993; Auld et al. 2016). An example of multimodal complex cues is the response of male fruit flies to conspecific rivals, with longer mating durations in male *Drosophila* spp. elicited following detection of mating rivals via combinations of three sensory modalities: song, smell and touch (Bretman et al. 2011a, b; Maguire et al. 2015).

Individual components within complex cues may also elicit a receiver response on their own and then interact to alter this response (i.e. ‘multiple signals’) or elicit a response only if perceived together (‘multicomponent signals’; Glossary). We not only focus here mainly on transmission of information between individual signallers and receivers of the same species but also briefly outline the collation of information from multiple signallers. Our emphasis is on the assessment of complex cues by the receiver, the resulting expression of social and reproductive plasticity and associated fitness benefits in the receiver. We do not extensively discuss here the adaptive benefits of complex cues to the signaller, and we make no assumptions about whether information transmission is active or passive. However, a comprehensive understanding of complex cues requires the full roles of signaller and receiver to be evaluated (Hebets and Papaj 2005).

We focus on complex cues in which the components are expressed simultaneously, or near simultaneously, during one reproductive event/encounter and which then directly initiate a receiver response (rather than effects on future phenotypes). However, it should also be noted that the timing of the delivery or perception of different cue components is important (Hebets and Papaj 2005). For example, the successful integration of multiple different cues expressed at different times, potentially across different reproductive events or life stages, may rely on learning or memory. In this way, individuals may employ past experiences and memory to inform their behaviour (Dukas 2006; Bailey and Zuk 2009). Hence, complex cues can also influence the learning and memory abilities of the receiver (‘receiver psychology hypothesis’; Table 1). A detailed investigation of learning in the expression of plasticity and the perception of temporally separated complex cues is reviewed elsewhere (Hebets and Papaj 2005).

**Table 1:** Hypotheses for the evolution of complex cues in animal communication, developed in the context of social/sexual plasticity. Evidence for the potential selective advantage of each hypothesis is given

Hypothesis	Theory	Evidence	Possible role in social/sexual plasticity
‘Backup signal’ ‘Redundant signal’	Multiple cues convey one message. The receiver benefits by assessing the message with increased accuracy. The signaller may benefit when the cost of signalling is reduced by spreading investment across multiple components (Møller and Pomiankowski 1993; Johnstone 1996).	Female swordtail fish distinguish hetero- and con-specific males more accurately based on both chemical and visual cues (Hankison and Morris 2003); male wolf spiders use more visual courtship displays when seismic components are inhibited (Gordon and Uetz 2011).	Improved robustness of information transmission in fluctuating social environments (Bro-Jørgensen 2010) and/or accelerated passing of a stimulus threshold (Rouse and Bretman 2016), resulting in phenotypes better suited to the current environment.
‘Multiple messages’	Each cue conveys a different message to one receiver. For example, different sexual ornaments could reflect different aspects of male quality. The signaller and/or the receiver may benefit by increasing the scope of information that can be exchanged (Møller and Pomiankowski 1993).	Components of great tit (*Xiphophorus pygmaeus*) birdsong are related to different measures of male quality (Rivera-Gutierrez et al. 2010); agonistic male-male signalling in eland antelopes (*Tragelaphus oryx*) reflect separate aspects of fighting ability (Bro-Jørgensen and Dabelsteen 2008).	Plastic responses can be fine-tuned to multiple features of the environment.
‘Unreliable signal’	Only one cue is a reliable indicator of quality. Any other signals are maintained because they are not costly to produce and are subject to weak Fisherian runaway selection (Fisher 1930). The signaller gains some benefit from the additional, more minor mate preference. The receiver does not gain any increase in the accuracy of the message (Møller and Pomiankowski 1993; Hankison and Morris 2003).	Bill brightness is significantly correlated with male mating success in mallards (*Anas platyrhynchos*) and plumage only loosely correlated (Omland 1996); female red jungle fowl (*Gallus gallus*) show a primary preference for male comb colour and weaker preferences for other ornaments (Johnsen and Zuk 1996).	No likely application to social/sexual plasticity.
‘Emergent message’	A single message emerges through the combination of non-redundant cue components. May benefit the receiver by conveying a more general and informative message based on multiple factors (Partan and Marler 2005; Bro-Jørgensen 2010).	Multiple species of songbirds account for a trade-off between trill rate and frequency bandwidth when assessing trills (Ballentine et al. 2004; Illes et al. 2006; Bro-Jørgensen 2010).	Plastic responses can be fine-tuned to multiple features of the environment, and information transmission is more robust.
‘Alerting signal’	One cue component may catch the attention of the receiver and direct it towards one or more other, informative signals. The signaller and the receiver may benefit from improved transmission of the message(s) (Hebets and Papaj 2005; Bro-Jørgensen 2010).	Bornean ranid frog (*Staurois guttatus*) calls direct the attention of conspecifics towards a visual foot-flagging display (Grafe and Wanger 2007); olfactory signals from male Gasterosteidae sticklebacks may make females alert to subsequent visual signals and increase detection (McLennan 2003).	Informative cues are made more salient, resulting in a phenotype better-matched to the social environment.
‘Receiver psychology’	Complex cues may benefit the signaller and the receiver by enhancing detection, discrimination, learning and memory of the message (Guilford and Dawkins 1993; Candolin 2003; Hebets and Papaj 2005; Bro-Jørgensen 2010).	The presence of auditory signals improves the speed of colour discrimination in domestic chicks (*Gallus Gallus domesticus*; Rowe 2002); audiovisual stimuli enhance song acquisition and quality in nightingales (*Luscinia megarhynchos*; Hultsch et al. 1999).	Informative cues are more salient, influential or efficiently processed; as in ‘alerting signal’.
‘Sensory overload’	In agonistic interactions, the signaller may benefit from the transmission of complex cues by reducing the accuracy and/or speed of message transmission (Hebets and Papaj 2005; Bro-Jørgensen 2010).	Dark-eyed juncos (*Junco hyemalis*) react more slowly when exposed to alarm calls in addition to a visual cue, compared to the visual cue alone (Randolet et al. 2014).	The response of the receiver may be rendered less advantageous or more costly due to time lags (Padilla and Adolph 1996; DeWitt et al. 1998) and phenotype-environment mismatches to the benefit of the signaller.

**Fig. 1:**
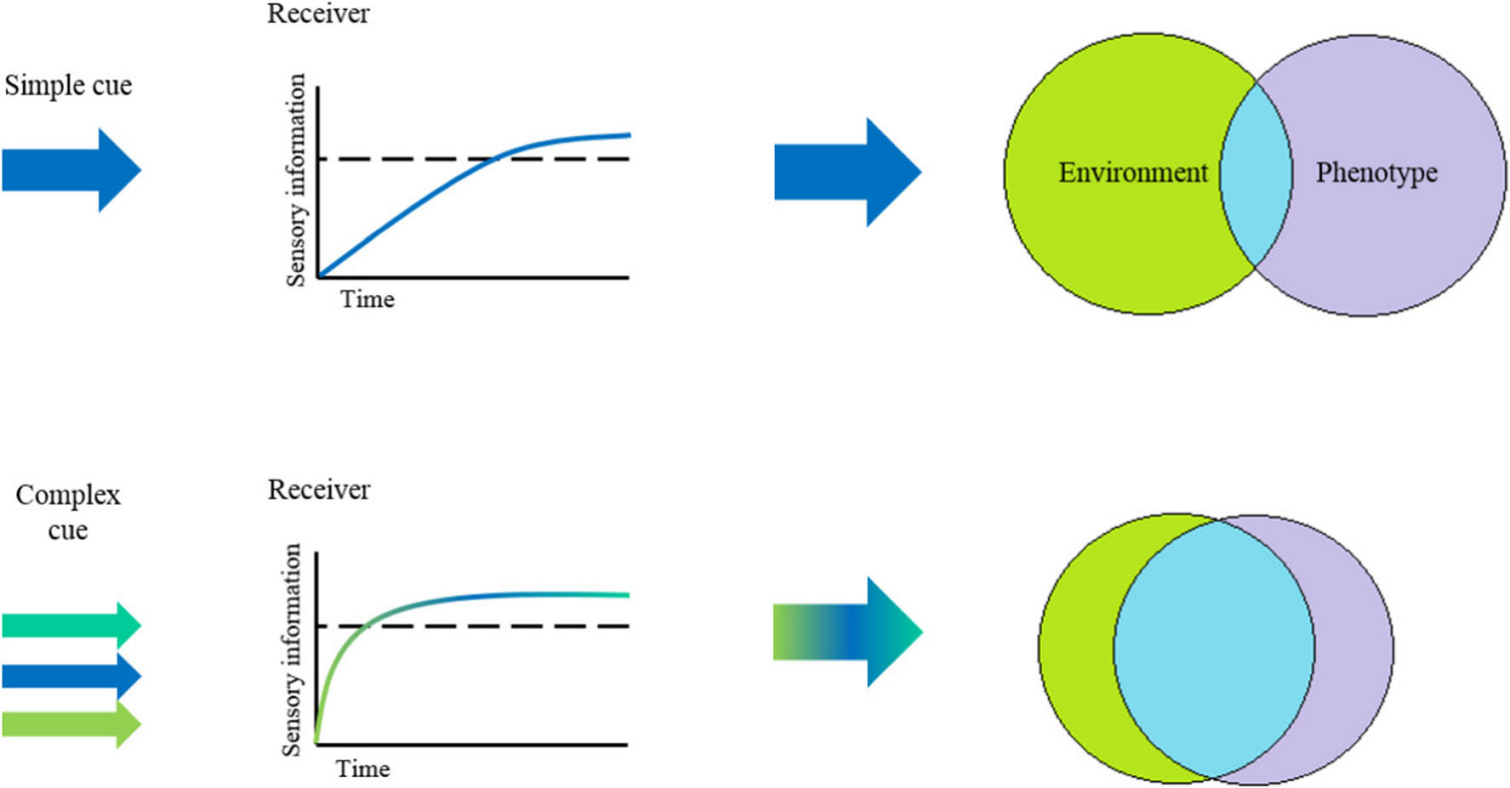
Complex cues reduce time lags. The perception of cues from the social and sexual environment comprising multiple distinct sensory components (complex cues in multiple colours versus simple cues in one colour) can decrease the time taken to reach sensory thresholds required to initiate a response (dotted line), resulting in a shorter time lag between environmental change and phenotypic change and hence a better-adapted phenotype

The perception of complex cues may play an important role in the expression of adaptive plasticity in response to varied and rapidly changing social and sexual environments (Charlat et al. 2007; Bretman et al. 2011a) according to factors such as the density of conspecifics and characteristics of competitors and mates (Oliveira 2012). Phenotypic plasticity is predicted to evolve in environments that vary rapidly, with intermediate to high predictability—where predictability refers to the degree to which a cue is correlated with future environmental conditions (Botero et al. 2015). Social environments may often meet these criteria, as they can be variable over a short timescale (Bretman et al. 2011a). Features of the future social environment (e.g. sperm competition) can often be at least partly predicted by current conditions (e.g. the presence of other males in the vicinity). Moreover, responses, such as choosing mates or strategically allocating reproductive investment are directly linked to fitness. Hence, complex cues might be particularly relevant in a social/sexual context if, as predicted, they can increase accuracy and/or speed of the response to environmental change and hence minimise the effects of mismatches in phenotypes directly linked to fitness (Charlat et al. 2007; Bretman et al. 2011a; Fig. 1).

Plastic responses can be modelled as a reaction norm—a function describing the expression of an individual’s phenotype across an environmental gradient (Via et al. 1995; see Nussey et al. 2007 for discussion of reaction norm approach). A plastic response can be characterised by the elevation (the degree of expression of the response) and the slope (the extent to which the expression of the response changes across the environmental gradient, i.e. the degree of plasticity). Individuals may vary in both the elevation and slope of a response due to genetic and non-genetic factors, such as experience and condition (Blumstein and Bouskila 1996; Nussey et al. 2007). Furthermore, social interactions are proposed to influence the evolution of reaction norms, affecting both between-individual and within-individual variation in the elevation and slope of a social response (see Dingemanse and Araya-Ajoy 2015). Theory on complex cues can be applied to reaction norms, with the environmental gradient of the reaction norm referring to the composite message that the complex cue confers. For example, an environmental gradient of potential sperm competition can be assessed through a complex cue of rival presence consisting of olfactory, auditory and tactile stimuli (Bretman et al. 2011b). Plastic responses to such gradients can occur on a spectrum of specialist to generalist (Gabriel et al. 2005). A more specialist strategy may be characterised by a steeper gradient, resulting in a highly expressed response in some environments and a low level of expression in others, whereas a generalist strategy may be modelled by a flatter reaction norm. Time lags between environmental change and response, and receiving incomplete information on the environment, are predicted to result in a trade-off between specialist and generalist strategies of reversible plasticity (Gabriel et al. 2005). As discussed below, complex cues may confer more complete environmental information and elicit faster responses, allowing for higher maximum fitness in the current environment at reduced cost to future fitness when the environment changes.

## Social and sexual plasticity is expressed by both sexes

Adaptive reproductive plasticity is expected whenever reproductive investment or resources are limiting. Individuals might respond to the presence, density, local sex ratio, quality or relatedness of conspecifics, and this may affect both behavioural and physiological investment in reproduction such as mate choice, displaying or mate guarding, fecundity, oviposition site choice or parental care (Glossary). We expect social plasticity to be common in females, because reproductive investment is often substantial in this sex. The empirical data support this view, with female mate choice often observed as highly socially plastic (Rodriguez et al. 2013; Lyons et al. 2014). Other female reproductive behaviours also exhibit social plasticity. For example, oviposition by *Drosophila melanogaster* females can be tailored to the social environment. When encountering a new site, females show a preference to oviposit in the presence of another female who has already laid eggs at that site (Sarin and Dukas 2009). However, it is becoming increasingly evident that males may also often exhibit reproductive plasticity. This is because, contrary to the traditional view that a male’s reproductive investment is much lower than that of females (Bateman 1948; Clutton-Brock and Parker 1992; Ahnesjo et al. 2001), it is now known that ejaculate production can incur considerable costs (Dewsbury 1982; Parker 1982; Wedell et al. 2002). Hence, a trade-off between investing in current and future mating opportunities can shape the optimum ejaculate transfer for any given reproductive episode and hence select for plasticity (Dewsbury 1982; Parker 1982; Wedell et al. 2002). There is abundant evidence that males can adapt their ejaculate investment to features of their social environment, for example, to the presence of rivals and to female quality (Wedell et al. 2002; Bretman et al. 2011a). This suggests that social plasticity in reproductive phenotypes can confer substantial benefits and conversely that fixed or ‘trial-and-error’ responses may result in lower fitness. However, in order for such plasticity to be adaptive, it should be based on accurate information (DeWitt et al. 1998; Auld et al. 2010). Hence, the role of complex cues in increasing the quantity and quality of social information, in comparison to simple cues, can be important for both sexes.

Complex cues may have a particular role in intersexual conflict. For example, there can be a selective advantage to males conveying deceptive cues to females regarding their individual quality, while females are selected to detect honest information (Holland and Rice 1998). As females evolve resistance to one deceptive male trait, males may evolve new cues to manipulate female perception of quality, resulting in multicomponent mating displays. In this way, sexual conflict has been proposed to promote the evolution of complex cues (Candolin 2003; Bro-Jørgensen 2010). Further research on the relationship between sexual conflict and complex cues is required to fully understand these dynamics.

## The importance of cues varies across the life course

The perception and processing of complex and simple cues, as well as the fitness consequences of social and sexual plasticity, are likely to vary across life stages (Groothuis and Taborsky 2015). The level of plastic responses can be fixed irreversibly during development (developmental plasticity), either in anticipation of the future environment (anticipatory plasticity) or as a response to the current conditions (reactive developmental plasticity; Kasumovic and Brooks 2011; Kasumovic 2013; Snell-Rood 2013). Alternatively, plasticity can occur as a rapid, flexible and potentially reversible response at any life stage (activational plasticity; Snell-Rood 2013). In activational and reactive developmental plasticity, the use of complex cues to produce faster responses (Fig. 1) may be particularly beneficial in avoiding time lags between cue detection and phenotype expression and hence poor environment matching. Complex cues may also be beneficial in providing more and higher quality information to fine-tune plastic phenotypes (Kasumovic 2013; Table 2). The way complex cues are processed, and the relative importance of the separate components for determining the response, may vary across different kinds of plasticity. Cue components that predict the future environment and/or are fairly stable over time may be more important for determining irreversibly plastic responses. In the case of relatively quick and reversible responses, cue components that fluctuate in relation to the immediate environment may be more pertinent.

**Table 2:** Hypotheses to explain the benefits of the use of complex cues and their underlying assumptions

Hypothesis	Underlying assumptions
1. Complex cues can prevent information loss in variable environments	(i) Simple cues are significantly compromised in variable environments. (ii) This loss of information Leads to phenotypes mismatched to the environment. The resulting deleterious effects reduce fitness and hence exert selection for processing complex rather than simple cues. (iii) Cue components can convey equivalent information and are interchangeable to the extent that the overall message is intact if one or more components are compromised.
2. Complex cues can fine-tune plastic responses based on multiple features of the environment	(i) Cue components provide at least partially different information. (ii) Perception of a greater quantity of environmental information results in a better phenotype-environment match.
3. Complex cues can reduce time lags between environmental change and response	(i) Sensory thresholds for initiating responses exist. (ii) Complex cue components additively or synergistically contribute to meeting these thresholds. (iii) The information transferred by each cue component is correlated. (iv) A fast-responding phenotype confers adaptive benefits. (v) These benefits outweigh potential costs of changing the phenotype in response to an ephemeral environmental fluctuation.
All the above	(i) Genetic and phenotypic variation in ancestral populations existed, upon which natural selection acted to promote the processing of complex cues. (ii) Organisms have the sensory and cognitive capacity to receive, process and integrate more than one cue component. (iii) The production of complex cues either Directly benefits the signaller, or occurs for other purposes and is co-opted by the receiver. (iv) A phenotype that is more closely matched to the social/sexual environment confers fitness benefits. (v) These benefits outweigh the potential costs of processing complex cues.

There are also specific periods of development, known as sensitive windows, during which phenotypes can be strongly influenced by the environment. The number, strength and consistency of complex cues may be particularly important in accurately translating this environment (Fawcett and Frankenhuis 2015), particularly if it is highly variable (Bro-Jørgensen 2010). The fitness consequences of receiving accurate and informative cues from the immediate environment may be higher during early life because an individual has less experience of the longer-term prevailing environment (Frankenhuis and Panchanathan 2011). Later in life, this may be less important; for instance, as the probability of re-mating in the future declines with age it may become more advantageous to invest heavily in current mating opportunities regardless of the environment (Rebar and Greenfield 2017). In this scenario, the perception of complex cues will become less advantageous with age as the fitness benefits of environment-phenotype matching diminish. Complex cues and the potential for cue redundancy might also become increasingly advantageous with age and increasing senescence in sensory perception and cognitive processing.

## The evolution of complex cues

To date, the evolution of complex cues has mostly been discussed in the broader context of animal communication. We explore here how the predominant hypotheses for the selective advantages conferred by the use of complex cues can also be applied to the context of social and reproductive plasticity (Table 1). The evidence to support these hypotheses in this new context is variable, and so we also suggest ways in which the perception and production of complex cues could evolve under these scenarios.

The ‘backup signal’ and ‘multiple messages’ hypotheses are currently the best known (Table 1; Johnstone 1996; Partan and Marler 2005; McElroy et al. 2007; Girard et al. 2015). They emphasise the predominance of redundant and non-redundant cues, respectively (Johnstone 1996; Partan and Marler 2005; Stynoski and Noble 2012). Redundancy could ensure that responses are based on robust information, while non-redundancy could increase the range of the environmental information available to inform optimal phenotype expression (Table 1). Evidence for the backup signal hypothesis comes from the expression of reproductive plasticity by male *D. melanogaster* in response to intrasexual competitors, via the perception of auditory, olfactory and tactile cues. Any two of these cues in combination, or all three, result in the same magnitude of response, implying redundancy or incomplete redundancy (Hypothesis 3 below; Bretman et al. 2011b). In the multiple message hypothesis (Box 1), each cue conveys a different message. This is supported in the context of reproductive plasticity by the example of the detection of pheromones by *D. melanogaster* males, in which separate cues signal female presence versus mating status, respectively (Siwicki et al. 2005; Lacaille et al. 2007).

Box 1 Mechanisms underlying signal integration

**Table:** 

Integral to understanding the ultimate consequence of social and sexual cues is resolving how the mechanisms mediating cues and behaviour control plastic responses to dynamic environmental information. Short-term plastic responses to transient stimuli may be achieved via different mechanisms than those underlying longer-lasting responses. For example, rapid responses may be achieved by switches in neural state and persistent responses by changes in gene expression (Winbush et al. 2012; Montague and Baker 2016). There is evidence for this from courtship suppression in *Drosophila melanogaster*, in which multiple signal components are integrated into pathways resulting in a plastic response. In this system, males exposed to a previously mated female for 1 h plastically reduce their courtship effort towards any female encountered in the next 2–3 h (Siegel and Hall 1979). The chemical signal 9-pentacosene, which indicates the presence of a female (Siwicki et al. 2005), is integrated with stimuli that signal the female’s recent mating status, via components of the male cuticular hydrocarbon profile, which are transferred to the female during courtship and copulation (Lacaille et al. 2007). These stimuli initiate the cyclic adenosine monophosphate (cAMP) pathway, which is associated with learning and memory and involves a molecular cascade in the mushroom bodies of the brain (centres of olfactory learning in *Drosophila*; Siwicki and Ladewski 2003; Keene and Waddell 2007; Montague and Baker 2016). In addition to these neurological effects, expression changes in genes associated with long-term memory of courtship rejection were observed 24 h after exposure to a mated female (Winbush et al. 2012). This suggests that longer-term plastic responses can be induced by differential gene expression when neural correlates of signals are continually reinforced. This example also exemplifies the integration of multiple signal components into one pathway, potentially leading to signal ‘thresholds’ required to generate a response being met more quickly (Griffith and Ejima 2009).	

It is likely that the backup signal and multiple messages hypotheses, and their associated benefits to plasticity, are not mutually exclusive. Cues could be partially overlapping in information content or may convey different information via alternative combinations (Johnstone 1996; Ay et al. 2007). Evidence for this idea comes from ornate tree lizards (*Urosaurus ornatus*), in which male quality, which can affect plastic responses of competitors and potential mates (Kolm 2001; Swierk and Langkilde 2013), is communicated by a complex cue of multiple morphological and behavioural characteristics. Some characteristics are correlated, indicating a repertoire of partially overlapping cue components (McElroy et al. 2007). This may confer benefits to the receiver in terms of the robustness of the cue and the range of information transmitted. Nevertheless, the possibility for multimodal cues to act in a redundant or compensatory way likely depends on flexibility in cue production and in the cue components that can initiate a receiver response. It is possible that this may impose substantial evolutionary constraints (Gray et al. 2014), an idea tested in *Teleogryllus oceanicus* field crickets, in which female choice is based both on male song and CHC composition. A flatwing male morph, unable to produce song, has recently evolved in some Hawaiian populations. However, there has been no concomitant increase in the attractiveness of cuticular hydrocarbons, suggesting that the reduced ability to attract females via acoustic cues is not compensated through other sensory modalities (Gray et al. 2014). Therefore, insights into the evolution of complex cues could be gained by considering a spectrum from fully redundant to fully non-redundant cues, as well as recognising that cues may combine in different ways to convey different messages (Ay et al. 2007).

Whether the benefits of receiving complex cues fall under the multiple messages or backup signal hypotheses may also depend on the extent to which the social/sexual environment is predictable (Botero et al. 2015). In scenarios where the future conditions are closely correlated with current cues, the selective pressure to receive redundant complex cues as ‘backup’ for cue components with poor predictive accuracy is likely to be weaker. On the other hand, receiving multiple, highly predictive cue components may increase the amount of environmental information on which a future phenotype can be based, as described by the multiple messages hypothesis. When the environment is moderately predictable, redundant complex cues may allow for robust information to be received and an appropriate response to be expressed, even if one or more cue component has declined in predictive accuracy.

Potential advantages to the signaller are also key to the evolution of complex cues (Table 1). For example, in mutualistic interactions when the fitness interests of the signaller and the receiver are correlated, both may benefit from the increased robustness of information derived from complex cues. On the other hand, if there is a conflict of interests between the signaller and the receiver, the perception of complex cues could also evolve in scenarios in which only the receiver benefits, if cues arising in another context are intercepted by receivers. For example, components of cuticular hydrocarbon profiles transferred from males to females during courtship and mating are likely to have evolved prior to the capacity of later-arriving males to detect such cues to infer a female’s mating status (Siwicki et al. 2005; Lacaille et al. 2007). Selection for such detection by late-arriving males would not depend on benefits to the female. Transferring information via complex cues may also evolve in situations in which the signaller, but not the receiver, benefits (sensory overload hypothesis; Table 1). This can occur if the receiver has a pre-existing sensory and/or cognitive capacity to detect and process cues that the signaller can exploit (Valkonen et al. 2014).

The integration of multiple cue components to inform a response to the social and sexual environment may be subject to cognitive constraints (Table 2). However, even apparently ‘simple’ organisms can achieve this, as such neurological integration of multiple sensory inputs appears common in insects (Wessnitzer and Webb 2006; Leonard and Masek 2014). In *D. melanogaster*, male plasticity in courtship effort (Box 1; Siwicki and Ladewski 2003; Keene and Waddell 2007; Montague and Baker 2016), responses to rival males (Rouse et al. 2018) and female mate choice and oviposition decisions (Dickson 2008; Joseph et al. 2009; Sarin and Dukas 2009; Joseph and Heberlein 2012; Lin et al. 2015) all use multicomponent sensory information, in various combinations, and all utilise common genetic and neural pathways and brain regions. These studies are revealing that disparate behaviours relying on different combinations of cues can utilise similar neural mechanisms. Thus, once evolved, the neural costs of novel plasticity may actually be relatively low.

Studying the perception and processing of complex cues, and plasticity itself, is complicated by the fact that the detection and discrimination of cues does not always lead to an observable response. In addition to the necessary fitness benefits and mechanisms for plastic responses to complex social cues to evolve, resource availability and trade-offs may limit when, and to what degree, a response is expressed. Furthermore, the assessment and decision-making processes linking the perception of cues to the expression of a response may vary depending on the characteristics and experience of the individual (Blumstein and Bouskila 1996). Variation in how cue perception translates to a response may occur both between and within individuals depending on sex, age, experience, state and genotype (Blumstein and Bouskila 1996). For example, there may be individual variation in sensitivity to particular cue components. Redundancy in complex cues could override this variation such that the same message is received regardless of which cue components are strongly or weakly perceived. Alternatively, under the multiple messages theory, a complex cue may confer a different meaning to a male versus a female, or a young individual versus an old individual, depending on the differential sensitivity of each individual to specific cue components.

## Hypotheses for the benefits of complex cue use in social and reproductive plasticity

The evolution of complex cue perception relies on the assumption that the resulting fitness benefits outweigh costs (DeWitt et al. 1998; Auld et al. 2010) and on the absence of cognitive and evolutionary constraints (Table 2). Costs incurred by signallers producing complex over simple cues could include greater energy expenditure, higher risk of predation and disease, and increased potential for eavesdropping (Hebets and Papaj 2005; Bro-Jørgensen 2010). Receiver costs are less well studied but are likely to be associated with the increased energetic and cognitive effort of processing a greater quantity of information (DeWitt et al. 1998). It is also possible that components of complex cues may be unreliable or even contradictory, leading to receiver costs associated with the potential to be misled about an environment.

There may also be instances where the perception of complex cues increases uncertainty about the environment, rather than reducing it (Munoz and Blumstein 2012). One potential example of this is when the components of a complex cue vary over different temporal scales. Under the multiple messages hypothesis, this may be advantageous in some scenarios by conferring multiple strands of information about features of the environment that similarly vary differentially over time. For example, a display signalling male quality may include fixed cue components indicating good genes and temporally variable signal components indicating current state, e.g. parasite load (Hebets and Papaj 2005). However, in some cases, the overall meaning of the message may depend on synchrony between cue components. When these cue components become decoupled, the message may be disrupted, and the optimum receiver response may not be expressed (Taylor et al. 2011). Even when cue components are in synchrony, if multiple components each convey environmental information with a margin of error, perceiving a complex cue may increase the potential for incorrect information to be perceived.

Despite these potential costs of receiving complex cues, the many examples of social and reproductive behaviour in which there are significant disadvantages of phenotype-environment mismatches (Gwynne and Rentz 1983; Preston et al. 2001; Presgraves et al. 2003; Bretman et al. 2013) suggest that such costs can be outweighed, giving a selective advantage to the perception of robust and informative complex cues (Candolin 2003; Hebets and Papaj 2005; McElroy et al. 2007; Bro-Jørgensen 2010; Bretman et al. 2011b; Auld et al. 2016). Whether the benefits of processing complex cues outweigh the costs is likely to depend upon the individual’s experience, the ‘missed opportunity’ cost of not responding to the environmental change and the features of the current social environment (Munoz and Blumstein 2012; Munoz 2015). For example, if a female first processes one cue component which indicates that a nearby male is likely to be a heterospecific, there would be little benefit to processing additional components to determine the quality of the male. However, if the first cue component indicated that the male was a conspecific, the benefits of synthesising additional cues to further discriminate the type of male, and subsequent effects on reproductive fitness, may outweigh the costs of further information processing. Thus, processing complex cues may result in a net fitness gain or loss, depending on individual and environmental factors (Munoz and Blumstein 2012; Munoz 2015).

Below, we explore further the benefits of receiving complex cues in facilitating the adaptive expression of social and sexual plasticity, leading to fitness benefits through the production of more robust phenotypes that can be fine-tuned more rapidly to the prevailing environment, thus avoiding the costs of phenotype-environment mismatches (Fig. 1).

### Complex cues can provide robust information in variable environments

In a social and sexual environment that varies significantly through time and space (Charlat et al. 2007; Kasumovic et al. 2008), complex cues have the potential to confer more robust information than is true for simple cues, because they can contain back-up components. They may also prevent information loss in variable environments, under the assumption that a simple cue cannot accurately convey information across all environments (Hebets and Papaj 2005; Kaczorowski et al. 2012; Cole and Endler 2015; Table 2). There are several scenarios in which the efficiency of simple cues could be compromised by environmental fluctuations. The first is when the content of the message to be transmitted is spatiotemporally variable, but the cue is fixed (Bro-Jørgensen 2010). For example, the horn length of male sheep (*Ovis aries*) can function as a fixed signal of quality to females, which may influence female mate choice. Because it is an indicator phenotype expressed at a fixed level, it best predicts reproductive success when climates and environmental conditions are stable across generations (Robinson et al. 2008; Bro-Jørgensen 2010). In the second scenario, simple cues cease to be informative if they converge. Consider a scenario in which male quality is indicated by a cue linked to a handicap (Zahavi 1975; Iwasa and Pomiankowski 1991). In favourable environments, the accuracy of the information conferred by this cue could be compromised because all individuals may have the resources to fully express the handicap, irrespective of male quality. Conversely, in poor environments, no individuals can express the handicap. Hence, the handicap ceases to be an informative indicator of quality and the simple cue carries no information. Finally, simple cues will convey only limited information if receiver preferences vary with the environment due to changes in the relevance or accuracy of cues or their assessment costs (Bro-Jørgensen 2010).

Under the above scenarios, individuals that receive simple cues would obtain incomplete or inaccurate information concerning the social or sexual environment. However, if complex cues are received, alternative cue components can be available for scrutiny even if one is compromised. A complex cue may also comprise various components, with some being particularly informative in different environments. This could allow receivers to more accurately track a range of environments (Lyons et al. 2014; Reparaz et al. 2014; Rhebergen et al. 2015). For example, in the swordtail fish *Xiphophorus multilineatus*, female choice seems to be based upon the component of a complex visual cue that is a better indicator of male quality under the environmental conditions to which the female has been previously exposed (Lyons et al. 2014). In this instance, the use of complex cues allows females to match their responses more tightly to their developmental environment. Moreover, in the green swordtail *X. helleri*, female choice, which uses multicomponent cues, is dependent on social context, with a female’s preference changing according to the range of male types with which she is presented (Royle et al. 2008). Thus, complex cues may enhance the benefits of plasticity by providing more accurate information concerning variable social environments, to which plastic responses can then be tailored with increased efficiency. The perception of complex cues is likely to be particularly important in cases where plasticity is expressed as a permanent change in an individual’s lifetime. These kinds of responses may have greater long-term fitness consequences (Fawcett and Frankenhuis 2015).

As well as enhancing the benefits of plasticity by allowing specific responses to variable conditions, the receipt of complex cues may avoid costly mismatches between phenotypes and fluctuating social environments. Bretman et al. (2011b) proposed that receiving complex cues may increase the reliability of information perceived, allowing plastic responses to be better suited to the social-sexual environment and thus avoiding ‘off-target’ reproductive investment. For complex cues to be selected, these ‘mistakes’ must impose significant fitness costs (Table 2). Evidence for such costs comes from the finding that prolonged high-level investment in reproduction under extended exposure to competition leads to sperm depletion, fewer later mating opportunities and shorter lifespan (Preston et al. 2001; Bretman et al. 2013). Thus, significant costs can arise if inaccurate or incomplete information on the social environment leads to an individual responding by increasing investment in current reproduction when it is not advantageous. Other costly mistakes can occur if incorrect assessments of cues lead to mating or attempted matings with the same sex, close relatives, individuals of a different species or with inanimate objects (Gwynne and Rentz 1983; Keller and Waller 2002; Presgraves et al. 2003). Accurate transmission of social information using complex cues can avoid these scenarios and reduce fitness costs.

The hypothesis that complex cues can prevent information loss when environments vary assumes that their components are equivalent or interchangeable to the extent that the message remains intact even if one or more of the components is compromised. Support for this idea comes from Bretman et al. (2011b) in which it was found that the removal of single cues indicating the presence of male rivals in *D. melanogaster* led to no apparent reduction in response from males, in comparison to scenarios in which males could detect a full sensory repertoire. Similarly, in eland antelopes (*Tragelaphus oryx*), while some components of male displays were non-redundant, three separate cue components act as backup signals of androgen-related aggression (Bro-Jørgensen and Dabelsteen 2008). In addition, several aspects of male mating displays are correlated and redundant in peacock spiders (*Maratus volans*; Girard et al. 2015). These studies support the hypothesis that complex cues benefit receivers through the robust transmission of information that would otherwise be compromised in variable environments.

### Perception of complex cues can fine-tune plastic responses based on multiple features of the environment

A second hypothesis is that the perception of complex cues provides fitness benefits by producing a phenotype that is better-matched to the social and sexual environment. This is due to the ability of complex cues to provide a greater volume of information about multiple environmental factors. The expression of such benefits relies upon the assumption that cue components relay at least some partially distinct information and that this greater range of social and sexual information leads the plastic responses of receivers to better match the environment (Table 2). There is some empirical support for this idea. As discussed above, female mating preference in *T. oceanicus* crickets is based both on song components and CHCs. These traits are not correlated in males and are likely to convey distinct information about condition (via song) versus genetic compatibility (via CHCs; Simmons et al. 2013). Similarly, male great tit (*Parus major*) mating displays consist of multiple components, some of which overlap in the information content, while others apparently communicate separate facets of male quality (Rivera-Gutierrez et al. 2010). These data show that complex cue components have the capacity to confer partially distinct strands of information. This will increase the range of environmental information that can be received in comparison to simple cues and influence the plastic responses of receivers. The increased information content of complex cues may explain why female swordtail fish (*Xiphophorus nigrensis*) have faster reaction times in response to males when presented with variation in multiple cue components (Reding and Cummings 2017). Furthermore, Bretman et al. (2011b) suggested that in order for *D. melanogaster* males to appropriately respond to the presence of rivals, information must be perceived regarding the species, sex and prevalence of potential rivals. This could be achieved by the processing of multiple cue components. Subsequently, it has been shown that interfering with one cue alters the magnitude of off-target responses to heterospecific males (Bretman et al. 2017), providing some evidence that multiple cues indeed enable better environmental matching.

Complex cues are likely to be particularly pertinent in scenarios where information needs to be gathered from multiple individuals in order to inform adaptive choice. For example, female ocellated wrasse, *Symphodus ocellatus*, use indicators of a male’s prior mating success and of the presence of other spawning females to inform their mate choice (Alonzo 2008). Information about the level of sperm competition (both risk and intensity) can be assessed through the presence of rival males together with information on the mating status of females. For example, *T. oceanicus* crickets alter their ejaculate in response to cues gathered directly from other males and indirectly from females. Experience of rival male song is reported to increase the number of viable sperm in the ejaculate (Gray and Simmons 2013), whereas sperm numbers decrease when males are exposed to females experimentally exposed to a blend of CHCs extracted from multiple males (Thomas and Simmons 2009). Likewise, *D. melanogaster* males use information from males and females to strategically invest in different components of reproductive behaviour, i.e. alterations to mating duration and courting effort (Box 1). Thus, the perception of complex social and sexual cues may be adaptive in allowing the reproductive behaviours of the receiver to be calibrated to the presence of potential mates and the risk and intensity of competition.

A well-studied example of the integration of multiple stimuli to inform a response is that of courtship suppression in *D. melanogaster* (Griffith and Ejima 2009). In this, complex cues comprising information on different features of the social environment are consolidated into one physiological pathway (Box 1), allowing the resulting response to be fine-tuned to these multiple information strands. In this way, complex cues have the capacity to convey a greater range of environmental information than is the case for simple cues, increasing the benefits of plasticity. These data do not necessarily indicate a ‘one-to-one’ association between cues and messages, but instead, owing to the interchangeability of cues, that information detected by different sensory modalities can be partially overlapping, or combined in a degenerate manner (Ay et al. 2007).

### Complex cues can reduce time lags between environmental change and response

Complex cues may enable more rapid responses to the environment, allowing individuals to better experience the benefits of short-term plasticity. As noted above, a male’s response to conspecific rivals in *D. melanogaster* is highly sensitive to the social environment. It is also fully reversible and exhibits the capacity to switch on and off several times over several days (Bretman et al. 2012). A contributing factor to this ability to respond to short-term changes in rival presence may be the use of complex cues in the detection of competitors (Bretman et al. 2011b). This idea relies on the existence of sensory thresholds that need to be exceeded in order to initiate a response (Fig. 1) and that complex cue components can additively or synergistically contribute to reaching these thresholds such that receiving a complex cue results in a faster response than is the case for a simple cue (Table 2; Partan and Marler 2005; Bretman et al. 2011b; Smith and Evans 2013). Consistent with this, there is evidence for the existence of sensory thresholds to initiate responses across species (Blaxter 1968; Page et al. 1998; Brown et al. 2006). Additive or synergistic effects of cue components have been observed on female choice in field crickets and wolf spiders (Scheuber et al. 2004; Uetz et al. 2009). Thus, a response threshold may be exceeded more quickly when there are multiple sensory components acting as an input, enabling individuals to adapt more rapidly to novel environments (Fig. 1). Studies on *D. melanogaster* rival responses support this, with males with only one sensory cue removed being able to extend mating duration after exposure to rivals, but requiring a longer exposure time to exhibit the same magnitude of response (Rouse and Bretman 2016). This is paralleled by studies of female mate choice in gryllid crickets, whereby mating decisions are accelerated by the combination of acoustic and chemical cues. In *T. oceanicus*, females respond more quickly to male song if they are simultaneously presented with male CHCs (Bailey 2011). Conversely, removal of the ability to detect CHCs in *Gryllodes sigillatus* increases the time taken for females to mount males (Ryan and Sakaluk 2009). These lines of evidence suggest an important role for complex cues in securing the benefits of a plastic phenotype.

Performing costly adjustments to an environmental stimulus that is too short term to allow considerable fitness benefits to be achieved may negate the advantages of a rapidly changing phenotype (Table 2). However, this will be avoided if there are minimum periods of exposure to stimuli required to elicit responses, as observed in male *D. melanogaster* responses to rivals (Bretman et al. 2010). Beyond an adaptive minimum period, time lags between environmental change and phenotypic adaptation can reduce or negate the benefits of plasticity (Padilla and Adolph 1996; DeWitt et al. 1998). Rapid responses to the environment may be particularly important in a social context due to the dynamic nature of social environments (Charlat et al. 2007; Bretman et al. 2011a). Thus, the potential for complex cues to reduce time lags could be an important factor in facilitating social plasticity.

## Conclusion

Social and sexual plasticity are ecologically important processes, whose benefits depend upon accurate and reliable monitoring of the environment. Complex cues may have a key role in facilitating social and sexual plasticity and maintaining associated fitness benefits, particularly in the context of dynamic environments that can otherwise disrupt signal transmission and hinder adaptive responses. We have outlined how this could occur via the availability of alternative signals when one is compromised, via the opportunity to integrate multiple strands of environmental information, via the reduction of time lags in adaptive responses and through the avoidance of costly ‘mismatches’ between phenotype and environment. It is clear that the investigation of the roles and evolution of complex cues will significantly enhance our understanding of the existence and pattern of plasticity. For example, if the existence of complex cues was not known, one would investigate the effects of simple cues in the detection of the social and sexual environment by individual males of *D. melanogaster* and conclude that none were responsible, hence that the correct cue had not yet been identified. Rather than being able to conclude that combinations of these cues are actually used by males (Bretman et al. 2011b), the search for mechanisms involved would become confused. Further research into the costs, benefits and evolution of complex cues is expected to provide a significant advance in our general understanding of adaptive plasticity, as outlined below.

### Measurement of the benefits and costs of receiving complex cues

There is evidence that complex cues convey benefits by preserving information transmission across variable environments and by producing receiver phenotypes better matched to the environment (Taylor et al. 2005; Wilgers and Hebets 2011; Rhebergen et al. 2015). Though the study of complex cues has begun in the context of social and sexual plasticity (Bretman et al. 2011b; Rouse and Bretman 2016; Rouse et al. 2018), the benefits and costs are yet to be fully experimentally explored, especially in longitudinal studies. Such data would improve our understanding of how the perception of complex cues to inform social and reproductive plasticity evolved. Experiments on the effects of the systematic manipulation of social and sexual cues on phenotypes and fitness could be carried out under a broader range of conditions and taxa to test the hypotheses proposed above. For example, determining the effects of individual condition (e.g. by varying nutrition; Mason et al. 2016) on the ability to process complex cues may shed light on when the costs of receiving complex cues become limiting. It would also be beneficial to measure genetic, phenotypic and environmental variation within the populations in which the perception of complex cues has evolved. This could be achieved through genetic or phylogenetic analysis or by using artificial selection. Such research would yield a greater awareness of the conditions in which the perception of complex cues to inform social and reproductive plasticity is advantageous and when and how the perception of complex cues should evolve.

### The effects of age and experience on the role of complex cues in social and sexual plasticity

The effect of age on the expression of plasticity has begun to be explored, and this has already yielded useful information on how the influence of environmental cues changes across life stages (Rebar and Greenfield 2017). Such an approach could also be adopted in the context of complex social and sexual cues to give a greater understanding of how individuals respond depending on their own intrinsic state as well as environmental factors. Studies of expressed phenotypes and associated fitness effects under systematic manipulation of sensory cues could be performed on individuals of different ages. Longitudinal studies could also be conducted to establish a longer-term picture of the effects of complex social and sexual cues. Furthermore, studies of the role of complex cues on how experience influences social and reproductive behaviour via learning and memory would be useful. One approach could be to expose individuals to simple or complex cues and monitor their future social and reproductive behaviour. This would advance our understanding of how the perception of complex cues interacts with experience, learning and memory to affect phenotypic flexibility on a longer-term basis.

### The effects of additional features of cue information on the expression of plasticity and associated fitness outcomes

In addition to considering the presence and effects of separate components of complex cues, other features of social and sexual information might also be important for understanding how social cues evolve and function. Research into the influence of multimodal versus unimodal complex cues on the expression and fitness effects of social and reproductive plasticity would be valuable. Identifying the effects of different sources of information might also be important, for example whether directed signalling versus incidental cues, or honest versus deceptive information, translate into differences in responses and fitness effects. This could increase the resolution of our understanding of how the features of social and sexual information influence phenotypic and fitness outcomes and hence provide a useful basis for classifying plasticity and predicting its effects. Experimental manipulations of signallers could also be useful to determine whether the signaller’s ability to detect the receiver influences the transmission of information and receiver responses.

### The benefits and costs to the signaller of the production of complex social cues

The role of both the signaller and the receiver should be considered to give a full understanding of the role and evolution of complex cues in social/sexual plasticity (Hebets and Papaj 2005). The costs and benefits of producing complex cues could be investigated by examining the fitness outcomes of individuals able to produce complex cues in a social interaction or manipulated so as only to produce simple cues. Potential costs could further be uncovered by examining condition dependence in the production of complex cues by manipulating nutrition.

## Glossary

Complex cue: — A package of information assessed by an individual within one social encounter, consisting of two or more distinct components, which can be either multimodal or unimodal (see below). Adapted from Hebets and Papaj (2005), but here, ‘cue’ encompasses both deliberate and incidental communication. Complex cues (‘signals’ of Hebets and Papaj (2005)) can be further subdivided as follows: • Multicomponent cue—a complex cue in which none of the components alone result in a receiver response, but do when received together. Generally unimodal. • Multiple cues—a complex signal in which each of the components alone can elicit a receiver response, but when received simultaneously, the response may differ (‘emergent message hypothesis’; Table 1) • Multiple traits—a complex cue in which one component confers species identity and another signals other information, such as individual quality.
Cue components: — The elements of a complex cue.
Multimodal: — A cue derived across multiple sensory modalities, perceived by multiple sensory systems.
Receiver response: — The plastic change in behaviour, physiology or morphology, initiated by a cue in the individual that receives it.
Simple cue: — Information assessed by an individual, consisting of a single component of information.
Social/sexual cues: — Cues relating to the social and sexual environment, encompassing information *directly* signalled by conspecifics or heterospecifics (such as individual quality signalled to potential mates) or *incidental* social information (such as the population density, or the physical characteristics of other individuals).
Social/sexual/reproductive plasticity: — Variable phenotypic responses to social and sexual cues, in an individual’s social behaviour, mating strategies, reproductive behaviour or morphology.
Unimodal: — A cue derived from within one sensory modality (e.g. smell) and perceived by one sensory system.
